# Identification of Transcriptional Heterogeneity and Construction of a Prognostic Model for Melanoma Based on Single-Cell and Bulk Transcriptome Analysis

**DOI:** 10.3389/fcell.2022.874429

**Published:** 2022-05-13

**Authors:** Zijian Kang, Jing Wang, Wending Huang, Jianmin Liu, Wangjun Yan

**Affiliations:** ^1^ Neurovascular Center, Changhai Hospital, Naval Medical University, Shanghai, China; ^2^ Department of Rheumatology and Immunology, Second Affiliated Hospital of Naval Medical University, Shanghai, China; ^3^ Department of Musculoskeletal Surgery, Fudan University Shanghai Cancer Center, Shanghai, China; ^4^ Department of Oncology, Shanghai Medical College, Fudan University, Shanghai, China

**Keywords:** malignant skin cutaneous melanoma, single-cell RNA sequencing, intra-tumoral heterogeneity, prognostic risk score, immunotherapy

## Abstract

Melanoma is one of the most aggressive and heterogeneous life-threatening cancers. However, the heterogeneity of melanoma and its impact on clinical outcomes are largely unknown. In the present study, intra-tumoral heterogeneity of melanoma cell subpopulations was explored using public single-cell RNA sequencing data. Marker genes, transcription factor regulatory networks, and gene set enrichment analysis were further analyzed. Marker genes of each malignant cluster were screened to create a prognostic risk score, and a nomogram tool was further generated to predict the prognosis of melanoma patients. It was found that malignant cells were divided into six clusters by different marker genes and biological characteristics in which the cell cycling subset was significantly correlated with unfavorable clinical outcomes, and the Wnt signaling pathway-enriched subset may be correlated with the resistance to immunotherapy. Based on the malignant marker genes, melanoma patients in TCGA datasets were divided into three groups which had different survival rates and immune infiltration states. Five malignant cell markers (PSME2, ARID5A, SERPINE2, GPC3, and S100A11) were selected to generate a prognostic risk score. The risk score was associated with overall survival independent of routine clinicopathologic characteristics. The nomogram tool showed good performance with an area under the curve value of 0.802.

## Introduction

Malignant skin cutaneous melanoma (SKCM) is a common life-threatening malignancy with high metastasis and mortality rates (Rastrelli et al., 2014). It accounts for approximately 4% of all skin cancer cases and is the most fatal subtype of skin cancer (Lin et al., 2021). The incidence of melanoma continues to increase worldwide, and more than 57,000 people died from melanoma in 2020 (Bray et al., 2021; Sung et al., 2021). Traditional treatments aim to relieve symptoms and reduce tumor burden, without much help for prolonging survival. Immunotherapy has been a breakthrough approach for metastatic melanoma, such as anti-cytotoxic T-lymphocyte-associated protein 4 (CTLA-4) and anti-programmed cell death protein 1 (PD-1) antibodies, which are based on the activation of the anticancer immune system (Marzagalli et al., 2019). Despite the improvement in the clinical efficacy of these immune checkpoint inhibitors (ICIs), 30–40% of melanoma patients do not respond to ICIs, and 20–30% of patients eventually relapse (Balch et al., 2009). Therefore, the 5-year survival rate for melanoma is relatively low, only about 15% (Enninga et al., 2017).

The differences in clinical outcomes and sensitivity to the drugs can be attributed to the heterogeneity of melanoma, which refers to the different genetic or molecular features in different melanoma tumors (inter-tumoral heterogeneity) or within the same tumor (intra-tumoral heterogeneity, ITH) (Simonsen et al., 2015; Grzywa et al., 2017; Moshe et al., 2020), which may be associated with tumor properties, such as the formation of the tumor-promoting microenvironment and the resistance to immune therapy (McGranahan and Swanton, 2017; Gay et al., 2021). The rare and unique tumor subtypes might be pivotal in determining disease biology (Gide et al., 2019). Therefore, a better understanding of the inter-tumoral heterogeneity and ITH and their impact on disease progression and therapeutic efficacy is essential to overcome treatment challenges in melanoma.

Previously, bulk RNA sequencing techniques have been used to classify molecular subtypes, monitor the treatment response, and identify new therapeutic targets (Zhao et al., 2020; Gay et al., 2021). However, they could not provide a high-resolution landscape of cellular components in the tumor ecosystem. Single-cell RNA sequencing (scRNA-seq) has become a powerful tool to characterize distinct functional states at single-cell resolution and has been used to explore the complexity of the tumor microenvironment (TME) and the ITH in many types of tumors (Patel et al., 2014; Tirosh et al., 2016; Puram et al., 2017). These findings have provided potential biomarkers for tumor treatment and risk stratification and laid the foundation for precision therapies. However, few studies have focused on the characteristics of ITH in melanoma and explored the impact of different subtypes on the prognosis and response to ICI therapy.

In this study, we utilized public scRNA-seq data to make a comprehensive analysis of the molecular characteristics, biological pathways, and transcription factor (TF) regulatory network of melanoma in an attempt to explore the prognosis and the impact of ICI therapy on each malignant subset and screen subsets that may contribute to the poor prognosis and resistance to immune therapy. In addition, we used the bulk RNA-seq data to establish a prognostic model to classify different risk groups and predict the clinical outcomes of melanoma patients, hoping that our findings could help identify the potential therapeutic targets and provide a strategy for precision medicine treatment of melanoma based on tumor heterogeneity, thus improving the survival of patients with melanoma.

## Results

### Identification of 12 Cell Clusters in Melanoma Using scRNA-seq Data

**FIGURE 1:**
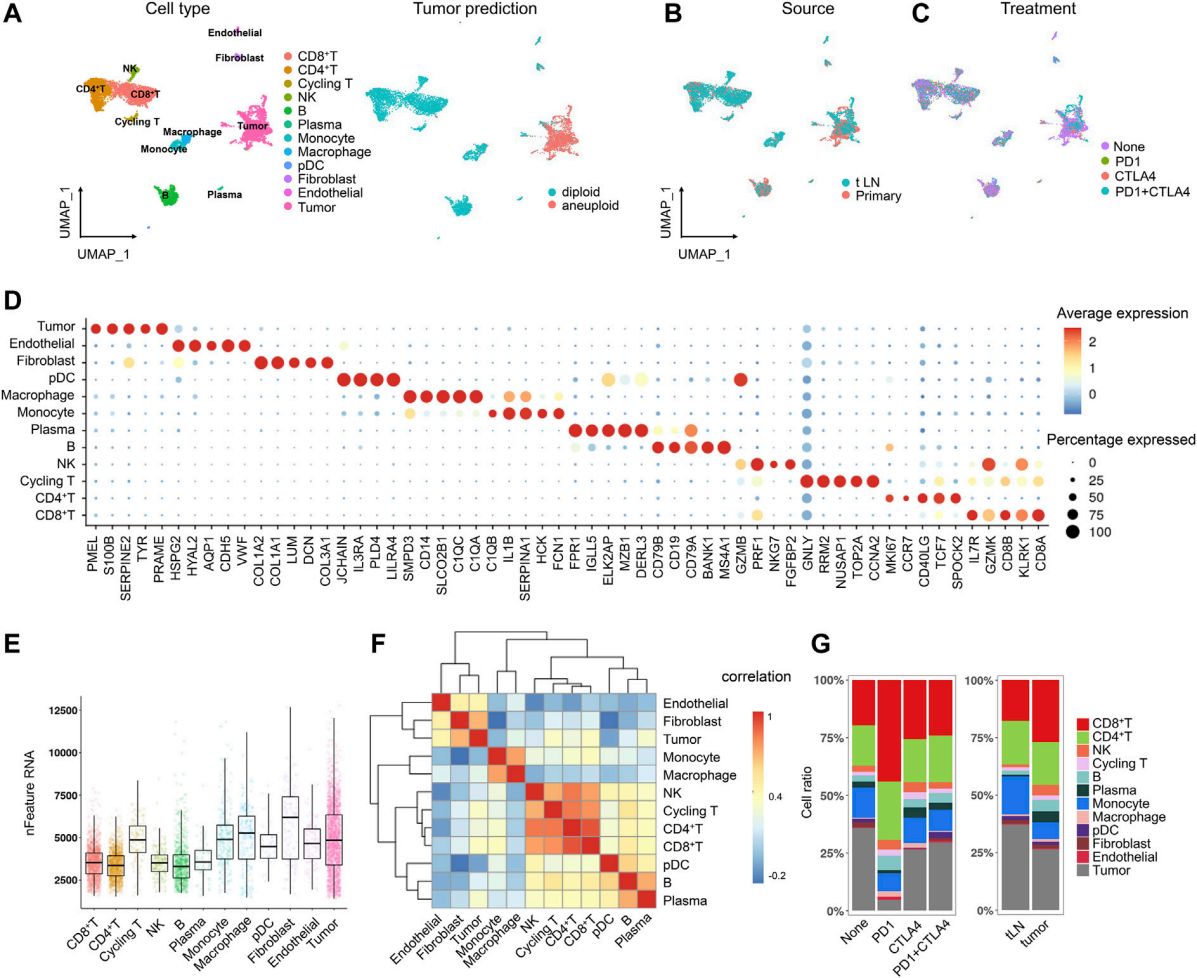
Single-cell atlas of the melanoma tissue. **(A)** UMAP projection of the landscape of melanoma; 12 main clusters were identified by transcriptome profiling across 7,186 cells after quality control, dimensionality reduction, and clustering. Tumor cells were predicted by CopyKAT **(B,C)**. UMAP projection of the clusters colored by sample source **(B)** and treatment **(C)**. **(D)** Dot plot displaying the fractions of expressing cells (dot size) and mean expression level in expressing cells (dot color) of marker genes (rows) across clusters **(E)**. Boxplot of the number of detected genes in clusters in melanoma **(F)**. Heatmap of the lineage correlations between melanoma T-cell clusters (CD4^+^ T, CD8^+^ T, NK, and T-prolif), B-cell clusters [**(B)**, plasma], and stromal clusters (endothelial cells and fibroblasts) were clustered **(G)**. Bar plots of the cellular sources for 12 clusters in different sample and treatment groups.

A total of 31 melanoma patients were involved in this study, including 15 untreated patients, 15 post-immunotherapy-resistant patients, and one was a post-immunotherapy responder. After the quality control (QC), we obtained 7,186 high-quality single-cell data, based on which we performed normalization, unsupervised dimensionality reduction, and graph-based clustering and finally obtained 12 cell clusters in the UMAP plot (Figure 1A). The cell types were annotated based on canonical known markers such as CD3D for T cells, CD79A for B cells, CD14 for monocytes, and DCN for fibroblasts (Supplementary Figure S1). Tumor cells were predicted by CopyKAT (Gao et al., 2021), an approach to identify genome-wide aneuploidy in single cells to separate tumor cells from normal cells (Gao et al., 2021). In addition to tumor cells, we identified nine immune cell lineages (CD45^+^), including natural killer (NK) cells, CD8^+^ T cells, CD4^+^ T cells, cycling T cells, B cells, plasma cells, monocytes, macrophages, and plasmacytoid dendritic cells (pDCs) along with two stromal cell lineages (CD45^−^), including endothelial cells and fibroblasts (Figure 1A). All the cell clusters were classified by sample source and treatment, showing no obvious batch effects in different groups (Figures 1B,C). The top five markers of each cluster were visualized in a bubble chart and were consistent with the typical markers (Figure 1D). PMEL, S100B, SERPINE2, TYR, and PRAME were highly expressed in tumor cells and could be the marker genes for melanoma (Figure 1D). Each cluster also differed in expressed gene counts (Figure 1E), and each lineage was clustered together, indicating a close lineage correlation among them (Figure 1F). These cell populations were distributed unevenly among treatment groups and tumor sites; however, due to the limited sample size, there was no statistical significance (Figure 1G).

### Inter-Tumoral and Intra-Tumor Heterogeneity in Melanoma Tumor Cells

**FIGURE 2:**
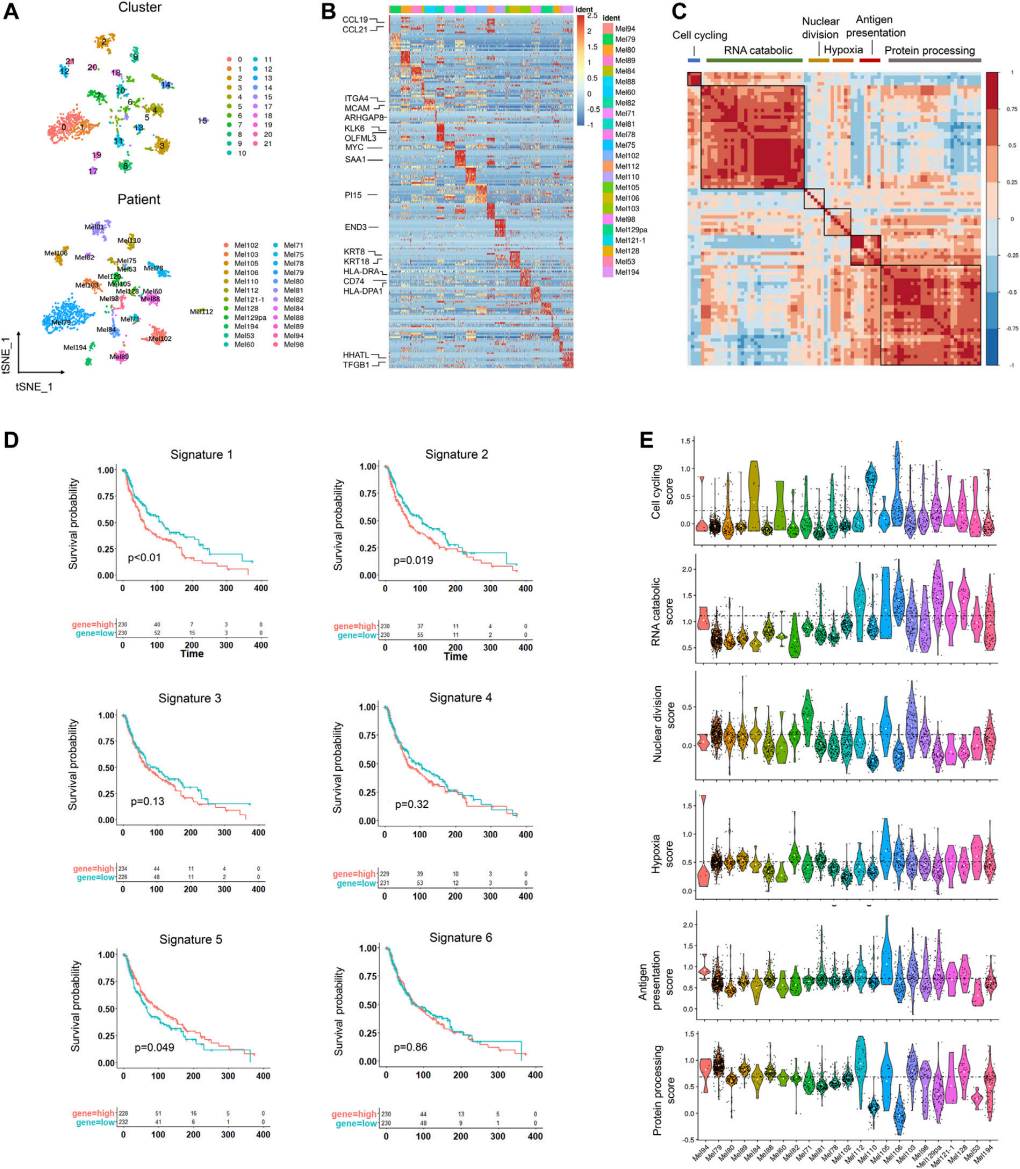
Inter-tumoral and intra-tumoral heterogeneity in melanoma tumor cells. **(A)** UMAP plot of 2089 malignant cells colored by clusters (upper) and patients (lower). **(B)** Heatmap showing the marker genes (rows) that are differentially expressed across individual tumors (columns), and selected genes are highlighted. The differentially expressed genes were calculated by the “FindAllmarkers” function in the Seurat package (logFC>0.25). The color of the heatmap represents the logarithmic scaled expression level of the genes. **(C)** Heatmap showing the pairwise correlations of metagenes derived from 22 tumors. Clustering identified six coherent malignant gene expression signatures across the tumors. **(D)** Kaplan–Meier curves for progression-free survival in the 460 patients in TCGA SKCM cohort according to high vs. low expression of the signature score evaluated by ssGSEA. The corresponding Cox regression *p* value is also shown. **(E)**. Violin plot showing the signature scores for one of the six malignant signatures for malignant cells from the 22 tumors.

To identify cell subclusters of tumors cells, we performed another round of normalization, unsupervised dimensionality reduction, and graph-based clustering and obtained a total of 22 clusters (Figure 2A). To detect differences between different patients, we classified the cells by patient origin and found that tumor cells were heterogeneous between different patients, suggesting a high degree of inter-tumoral heterogeneity (Figure 2A). Some patients also showed ITH, for example, Mel194, Mel105, and Mel78 contained multiple clusters. We then calculated the differentially expressed genes (DEGs) between patients and found that DEGs were enriched within pathways that varied across tumors, showing significant phenotypic diversity (Figure 2B, Supplementary Table S2). CCL21 and CCL19 were highly expressed in Mel94, which assisted in immunotherapy of cancers by potentiating immune response (Salem et al., 2021). KRT8 and KRT18 were highly expressed in Mel106, and they were extensively used as diagnostic tumor markers. Several studies have demonstrated their involvement in cancer cell invasion and metastasis as well as in treatment responsiveness (Figure 2B) (Karantza, 2011). Copy number variations (CNVs) are universal prognostic markers and established the concept of natural selection to drive carcinogenesis and acquired therapeutic resistance (Andor et al., 2016). Melanoma patients also had different CNVs, further validating the inter-tumoral heterogeneity in melanoma tumor cells. The distinct CNVs may determine the survival prognosis and drug treatment response for melanoma patients (Supplementary Figure S2).

To identify the common expression programs that varied within multiple tumors, we then applied nonnegative matrix factorization (NMF) (Gaujoux and Seoighe, 2010) to reduce dimension and identified a total of 3,190 metagenes that were preferentially co-expressed by subpopulations of malignant cells across tumors. Next, hierarchical clustering was applied to characterize these metagenes into gene expression signatures, and high concordance was shown in six signatures, indicating that they reflected common patterns of intra-tumoral expression heterogeneity (Figure 2C).

Of these signatures, the first signature was associated with cell cycle genes such as CDK1 and TOP2A. The second signature was related to the RNA catabolic process, and the third was enriched in nuclear division. The fourth signature reflected a hypoxia signature in tumor which may promote melanoma progression (D’Aguanno et al., 2021). The fifth signature contained genes associated with antigen presentation, which may help the response to checkpoint therapy. The last one was involved with protein processing (Figure 2C, Supplementary Figures S3A,B). We then estimated the prognostic significance of the six signatures and found cell cycling and RNA catabolic program were associated with poor prognosis, while the antigen signature was correlated with better survival time (Figure 2D). These signature scores also varied across the tumor cells from different tumors, suggesting that these signatures could characterize different tumors (Figure 2E).

### Subclustering of Melanoma Malignant Cells Based on Intra-Tumor Transcriptional Heterogeneity

**FIGURE 3:**
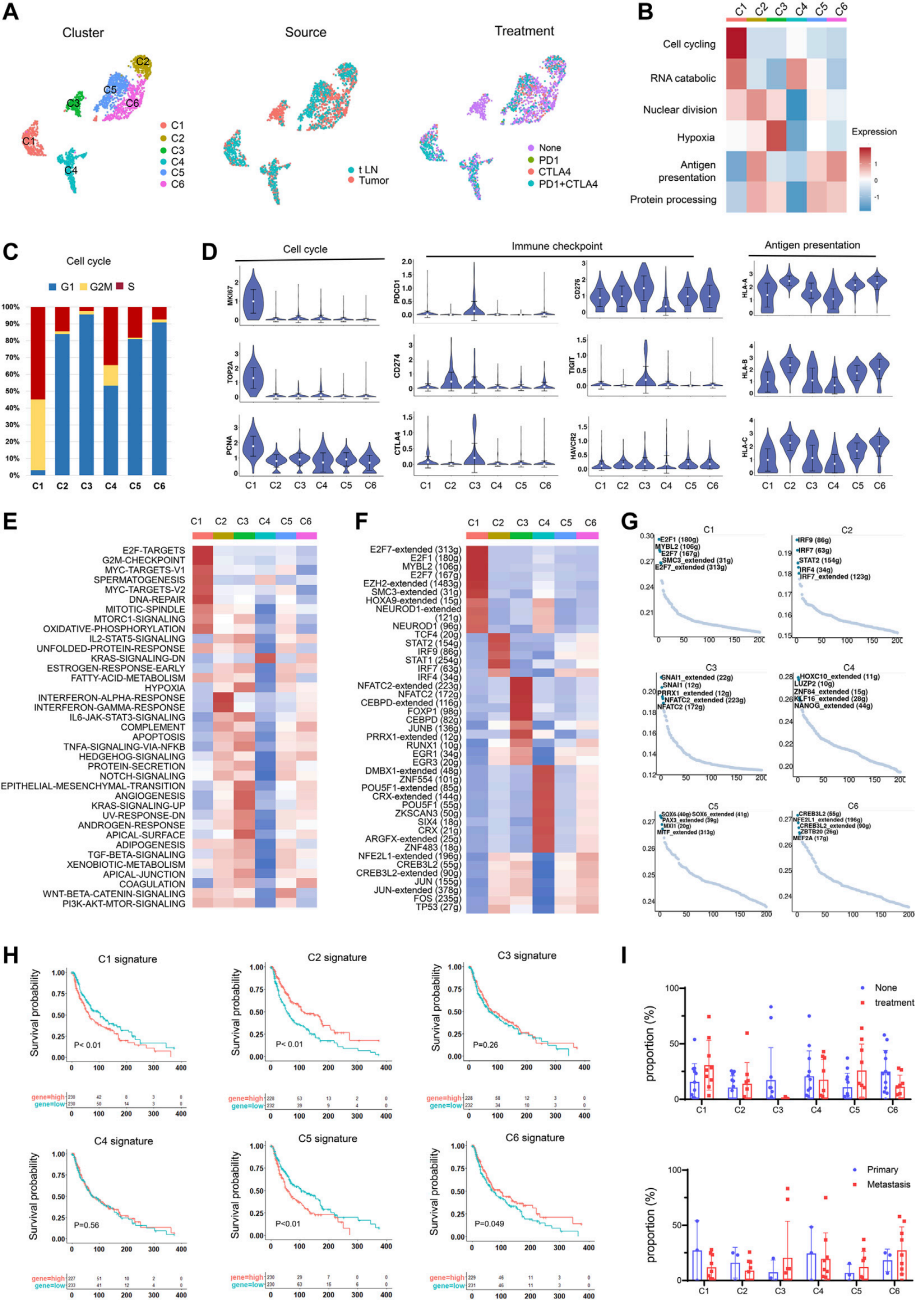
Identification of tumor cell subtypes. **(A)** UMAP plot showing the six subgroups generated from tumor cells. Cells were further shown in different colors by patient origin. **(B)** Differences in the malignant signature scored by ssGSEA between tumor subsets. **(C)** Bar plots showing the fraction of cell cycle (G1, G2M, S) in tumor subsets. **(D)** Violin plots of the expression levels of cell cycle genes, immune checkpoint genes, and the HLA genes in tumor subsets. **(E)** Differences in the hallmark gene set scored per cell by GSVA in tumor subsets. **(F)** Heatmap of the t values of AUC scores of expression regulation by TFs, as estimated using SCENIC for the cluster. t values from a linear model for the difference between cells from one cluster and cells from all other clusters, corrected for the patient of origin, and all TFs, are shown. Numbers within brackets indicate the (extended) regulons for respective TFs. **(G)** TF motif variability analysis in tumor subsets. The dot plot shows the rank-sorted TF motifs according to the specific score of TFs. **(H)** Kaplan–Meier curves for progression-free survival according to high vs. low expression of the cluster signature score evaluated by ssGSEA. **(I)** Proportions of tumor subsets in primary and metastatic tumors and non-treatment group and treatment group.

To identify subtypes of melanoma, we clustered tumor cells using the common patterns of intra-tumoral transcriptional heterogeneity from the metagenes. Six subsets were identified and were colored by the sample source and treatment (Figure 3A, Supplementary Figure S3C). The marker genes of the six subsets are shown in Supplementary Figure S3D; Supplementary Table S3. The identified signatures were preferentially, but not exclusively, co-expressed by subsets of tumor cells, for example, cluster 1 has high levels of cell cycling signature, cluster 3 is enriched for functions such as hypoxia signature, and cluster 6 has elevated levels of antigen presentation signature (Figure 3B). An overview of the cell cycle of each cluster showed cluster 1 has the highest proportion in the G2M/S stage (Figure 3C).

To further explore the characteristics of tumor subsets, we compared the expression of cell cycle genes, immune checkpoint genes, and antigen-presenting genes and found that cluster 1 showed high expression levels in cell growth genes, indicating an aggressive cluster in melanoma (Figure 3D). Cluster 3 highly expressed genes associated with immune checkpoints, which may indicate benefits from immunotherapy. Cluster 2 had an elevated antigen-presenting capacity and had highly expressed HLA molecules, which may suggest a favorable prognosis. The immunotherapy also had effects on gene expression. In most tumor subsets, we observed the upregulation of proliferation genes and downregulation of immune checkpoint genes and HLA molecules (Supplementary Figure S4), possibly due to the death of tumor cells with low proliferation and high expression of immune checkpoint genes and HLA genes after immunotherapy. The remaining tumor cells with high expression of proliferation genes and low expression of immune checkpoint genes and HLA molecules may cause resistance to immunotherapy or promote disease progression.

We next performed gene set variation analysis (GSVA) (Hänzelmann et al., 2013) to compare their biological functions (Figure 3E). Cluster 1 was enriched for “cell cycle,” “DNA repair”, and “mTOR signaling”, which facilitated tumor initiation, survival, and exacerbation. Cluster 2 was associated with IFN-γ and IFN-α responses. They might be successfully attacked by the evading T cells by the high expression of HLA molecules. Cluster 4 showed a relatively low biological function, suggesting a quiescent state. Cluster 3 showed high expression associated with hypoxia and angiogenesis. Hypoxia could induce metabolic and molecular changes in endothelial cells, thus increasing the expression of pro-angiogenic molecules and blood vessel formation (Muz et al., 2015). Cluster 5 showed elevated levels of Wnt signaling and fatty acid metabolism activity, which were correlated with melanoma progression and metastasis as well as response to targeted therapies (Alkaraki et al., 2021).

We further applied single-cell regulatory network inference and clustering (SCENIC) (Aibar et al., 2017) to explore the TF regulatory network in malignant subsets. Heatmap analysis of the top-ranking activity of TFs revealed different transcriptional regulation characteristics in malignant subsets (Figure 3F). For instance, the E2F7, E2F1, and MYBL2 were enriched in cluster 1, while the STAT1 and STAT2 were enriched in cluster 2. Cluster 3 has elevated activity of JUNB and CEBPD regulons, which were inflammation-responsive TFs. TF motif variability analysis also validated the specific TFs in each cluster (Figure 3G).

To identify cell subsets associated with distinct clinical outcomes, we calculated the signature score of each cluster and compared the survival time between the high- and low-expression groups. Clusters 1 and 5 were found to be associated with unfavorable outcomes and short survival time, and clusters 2 and 6 suggested better prognosis and longer survival of melanoma patients (Figure 3H). We further explored the effect of immunotherapy on the ratio of tumor subsets (Figure 3I). In immunotherapy-resistant patients, cluster 3 showed a decreasing trend after immunotherapy, possibly due to the high expression levels of immune checkpoint genes. Clusters 1 and 5 showed an upregulating trend after immunotherapy. The insensitivity of Clusters 1 and 5 to immunotherapy could be an important cause of immunotherapy resistance. Concerning the highly proliferative properties, the existing cluster 1 after ICI treatment may contribute to melanoma recurrence.

### Transcriptomic Signatures of Resistance to Checkpoint Blockade

**FIGURE 4:**
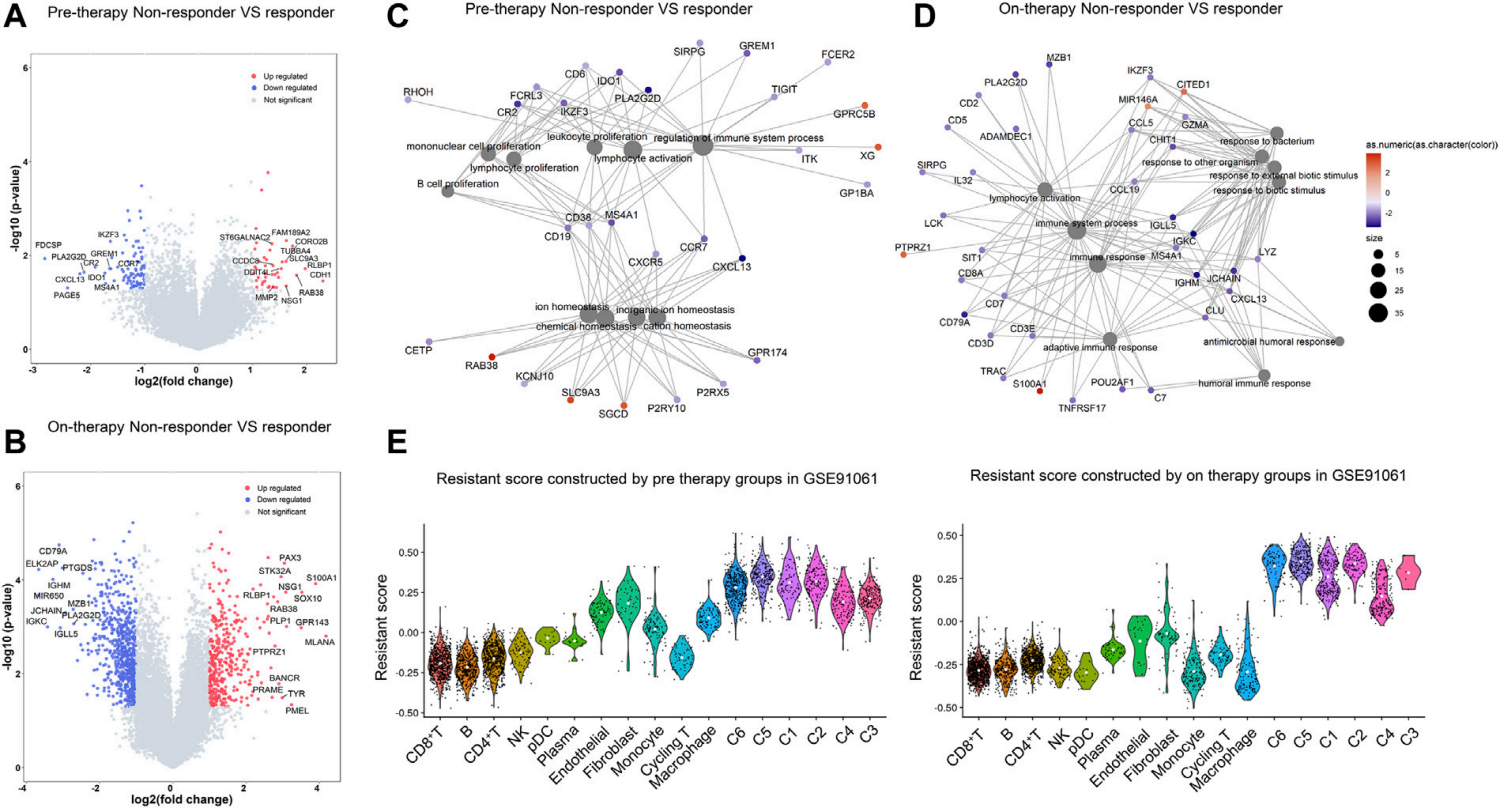
Transcriptomic signatures of resistance to checkpoint blockade. **(A,B)**. Differential gene expression between non-responders and responders in melanoma in the pre-therapy group **(A)** and on-therapy group **(B)**. Volcano plot showing differentially expressed genes in non-responders compared with responders according to the fold change (*x*-axis) and log *p* value (*y*-axis). The selected highly expressed genes [*p* value < 0.05 and a log2 (fold change) > 1] in Inf-salivary gland epithelial cells are labeled in red, and downregulated genes [*p* value < 0.05 and a log2 (fold change) < -1] are labeled in blue. **(C,D)**. GO enrichment network in non-responder patients in pre-therapy **(C)** and on-therapy group **(D)**. Significant differentially expressed genes (*p* < 0.05, |logFC|>1) were calculated between pSS and HCs. The color labeled on each gene indicates the log-fold change value. The bubbles connected with the genes were the enriched biological functions. The color labeled on bubbles suggests the *p* values. **(E)**. Resistant score to checkpoint blockade in single-cell clusters in pre-therapy and on-therapy groups. The resistant score was calculated based on the differentially expressed genes between non-responders and responders in melanoma in pre-therapy and on-therapy groups.

To identify the role of each cell cluster in resistance to immune checkpoint blockade, we analyzed the bulk-RNA data from the research by Riaz et al. (2017), which contained groups of different response patients including complete response/partial response (CR/PR), stable disease (SD), and progressive disease (PD) before and during immune therapy in melanoma patients. CR/PR and PD groups represent two opposite outcomes of immunotherapy. To obtain reliable immunotherapeutic response-related genes, we calculated DEGs between non-responders (PD) and responders (CR/PR) in melanoma in pre-therapy and on-therapy groups. In both pre-therapy and on-therapy groups, non-responders had low expression of immune cell-associated genes such as CCR7, CXCL13, MS4A1, CD79A, MZB1, and JCHAIN (Figures 4A,B). Non-responders highly expressed CDH1, KRT17, KRT14, and MMP2 in on-therapy groups, while had high expression of PMEL, TYR, S100A1 and SOX10 in on-therapy groups. The DEGs were enriched in immune response pathways such as “lymphocyte activation,” “regulation of the immune system process,” and “adaptive immune response,” suggesting that the abundance of infiltrating immune cells was correlated with the response to ICI treatment (Figures 4C,D).

We then computed the resistance score of each cluster to evaluate the response to ICI treatment by using the differential gene expression patterns in pre-therapy and on-therapy groups (Method). Malignant clusters showed higher resistant scores than other cell types, and cluster 5 had the highest score in pre-therapy groups and on-therapy groups (Figure 4E). Furthermore, cluster 5 was correlated with short survival time and had a high proportion after ICI treatment (Figures 3H,I), which could be a drug-resistant tumor cluster. In non-malignant cells, CD8^+^ T and B cells had the lowest score, which was a benefit to ICI treatment. Fibroblast and endothelial cells had high resistant scores, suggesting stroma cells could confer resistance to immune-based therapies for melanoma.

### Consensus Clustering for Malignant Genes Correlated With Melanoma Prognosis and Immune Microenvironment

To further investigate the clinical value of tumor heterogeneity, TCGA SKCM patients were divided into three different subtypes using the metagenes with ConsensusClusterPlus (Wilkerson and Hayes, 2010) (Supplementary Figure S5A). Compared with the patients from clusters 1 and 2, patients in cluster 3 showed a significantly worse outcome (Supplementary Figure S5B). ssGSEA analysis of the tumor microenvironment in three groups showed that patients in cluster 3 had high tumor purity and low immune infiltration, corresponding to the immune desert tumor. Patients in cluster 1 showed high tumor purity and relatively high immune infiltration, which may be sensitive to immunotherapy. Patients in cluster 2 showed low tumor purity and relatively high immune infiltration, which may indicate good prognosis (Supplementary Figure S5C). The functional enrichment analysis further confirmed that patients in cluster 3 were related to tumor progression pathways such as “fatty metabolism,” “DNA repair,” and “cell cycle” (Supplementary Figure S5D). They also showed low activity in immune responses such as IFN-γ, TNF, and inflammation response as well as IL2, IL6, and TNF signaling. These pathways were upregulated in cluster 2 patients (Supplementary Figure S5D). Taken together, the tumor metagenes could effectively distinguish the tumor characteristics and tumor microenvironment in SKCM patients and were critical for the stratification of melanoma patients.

### Construction of Risk Signature for Melanoma Survival

**FIGURE 5:**
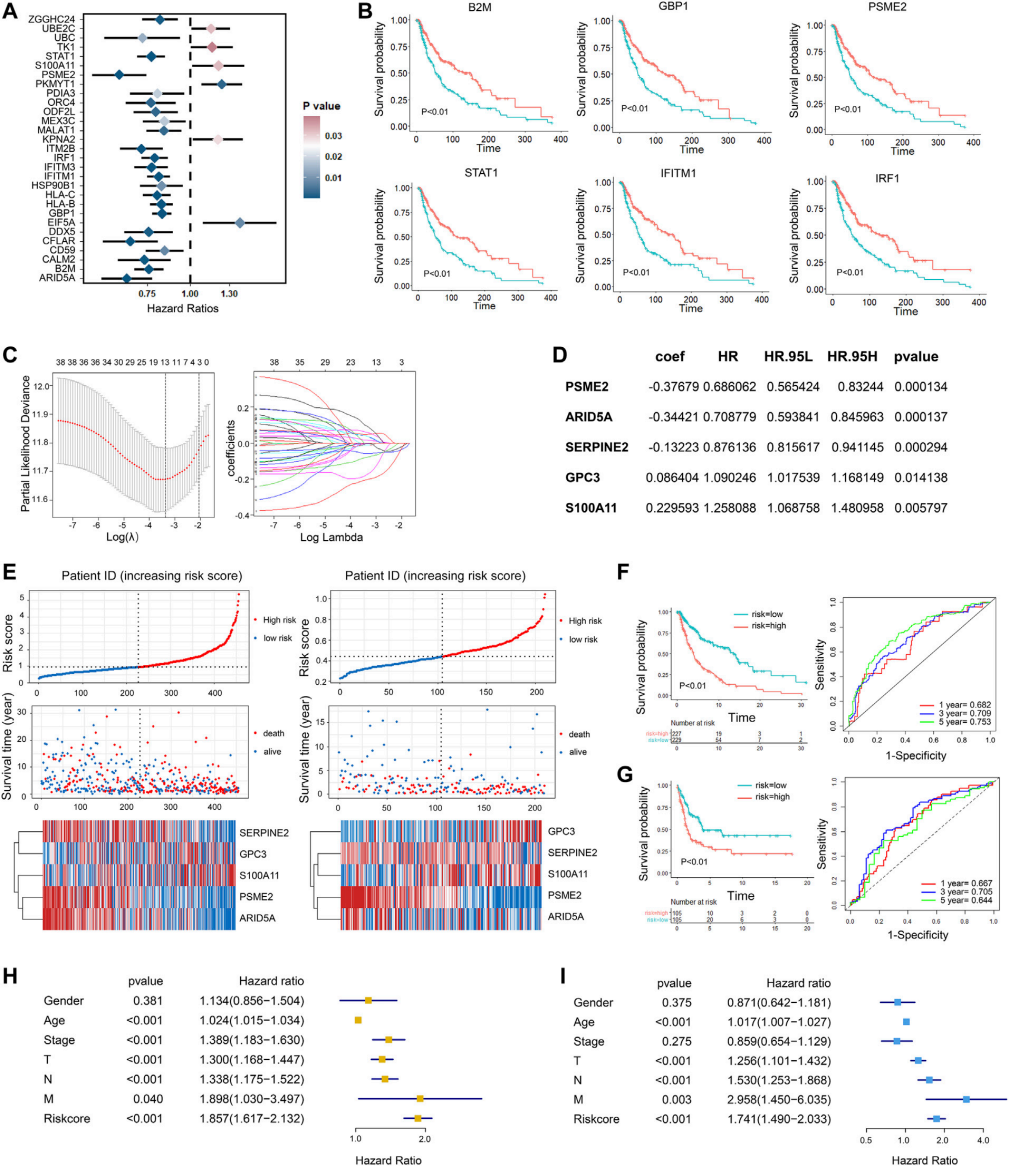
Development of gene signatures for overall survival prediction in melanoma patients. **(A)** Univariate Cox regression analysis showing the hazard ratios (HRs) with 95% confidence intervals (CIs) and *p* values for metagenes. **(B)** Kaplan–Meier curves for progression-free survival of the representative genes identified by univariate Cox regression. **(C)** Cross-validation for tuning parameter screening in the LASSO regression model. **(D)** The five genes’ hazard ratios (HRs) and 95% confidence intervals (CIs) and coefficients screened by multivariate Cox regression. **(E)** The distribution of the risk score and survival overview of melanoma patients in TCGA cohort (left) and validation cohort (right). In each dataset, the risk score distribution, gene expression profiles, and patients’ survival status are displayed. **(F,G)**. Kaplan–Meier and time-dependent ROC analysis for the risk score in the training cohort **(F)** and validation cohort **(G)**. **(H,I)** Univariate Cox **(H)** and multivariate Cox **(I)** regression analyses of clinical parameters and prognostic model for OS.

Considering the prognostic value of subset marker genes in patients with melanoma, we intended to construct a risk score model to evaluate the prognosis status of melanoma patients more accurately. First, we performed univariate Cox regression analysis with the top 30 marker genes in each tumor cluster and screened out 41 genes that were significantly associated with the prognosis of melanoma patients in TCGA dataset (*p* < 0.05, Supplementary Table S4), and the representative genes are shown in Figure 5A (HR <=0.85 or HR>= 1.15). The survival state of the most significant genes is shown in Figure 5B. After that, we performed the lasso Cox regression analysis to screen the most valuable predictive genes (Figure 5C). After hypothesis testing, five genes were selected with a *p*-value of less than 0.05, and the risk signature was constructed by multivariate Cox analysis (Figure 5D). Patients in TCGA SKCM cohort and GSE65904 (Cirenajwis et al., 2015) cohort were divided into high-risk and low-risk groups based on risk scores. Compared with those in the low-risk group, the melanoma patients in the high-risk group had high expression of S100A11 and CPC3 and low expression of PSME2, ARID5A, and SERPINE2 and had a shorter survival time (Figure 5E). The time-dependent receiver operating characteristic (ROC) curves revealed that the area under curve (AUC) of 1-, 3-, and 5-year survival in TCGA was 0.68, 0.71, and 0.75, respectively, vs. 0.67, 0.71, and 0.64 in GSE65904 cohort (Figures 5F,G). Cox regression analysis was used to further investigate the clinical prognostic significance of the risk signature in melanoma. The univariate analysis showed that the risk score, age, and TNM stage were significantly associated with survival time (Figure 5H). After adjusting these factors in the multivariate analysis, we found that the risk signature was still a significant survival predictor, indicating that the risk signature was independent and not affected by other factors such as age and gender (Figure 5I).

### Construction of Nomogram for Melanoma Survival

**FIGURE 6:**
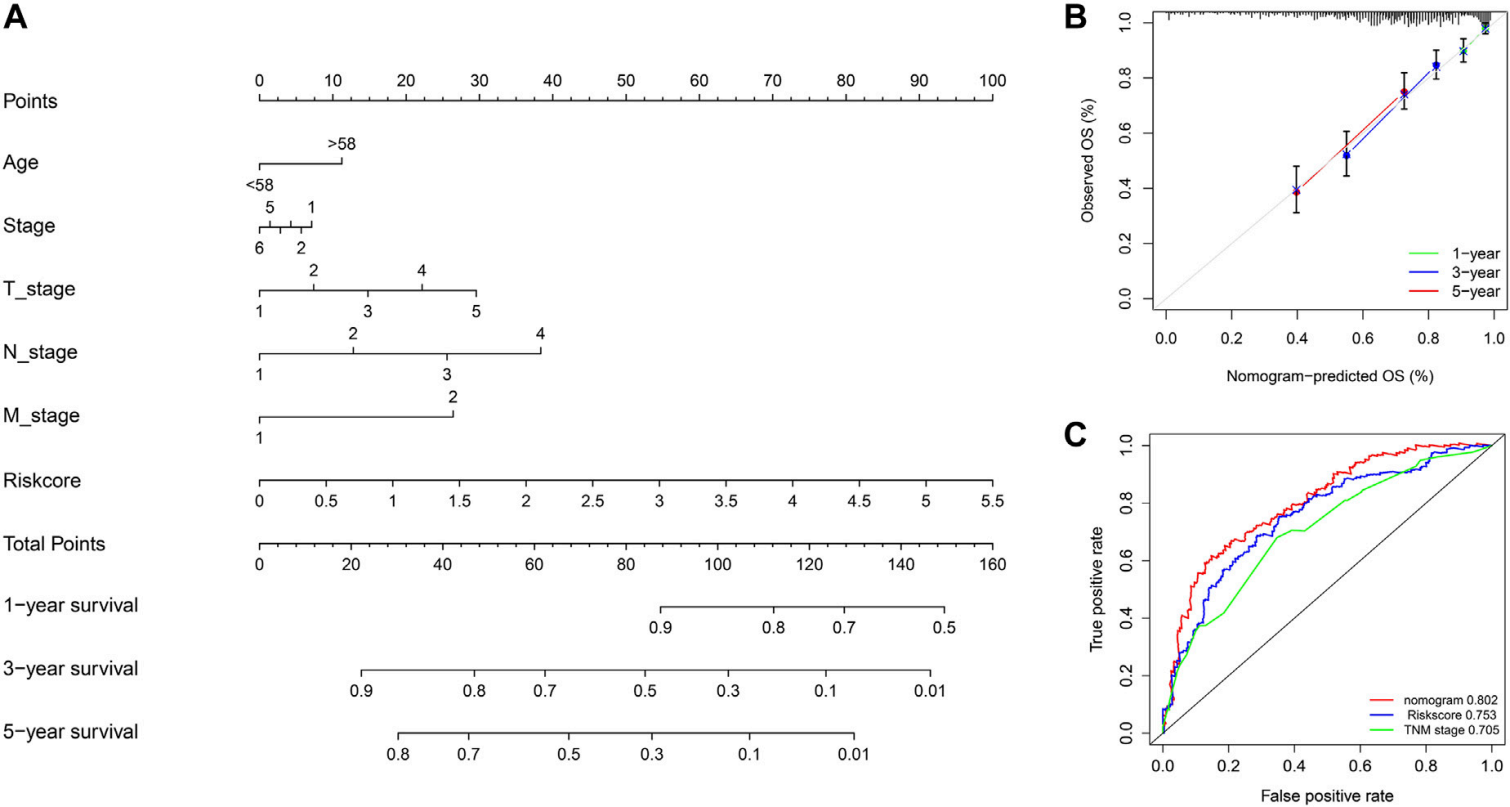
Nomogram model for predicting the overall survival of melanoma patients. **(A)** Nomogram for predicting 1-, 3-, and 5-year overall survival for melanoma patients in TCGA cohort. **(B)** Calibration curves of nomograms in terms of the agreement between predicted and observed 1, 3, and 5 years of outcomes in TCGA cohort. **(C)** Time-dependent ROC curves comparing prognostic accuracy of risk score, TNM stage, and the nomogram model in TCGA SKCM patients.

To predict the prognosis of melanoma patients more accurately, we established a prognostic nomogram to predict the survival probability at 1, 3, and 5 years based on TCGA training set. Independent prognostic parameters, including age, TNM stage, and risk score, were enrolled in the prediction model (Figure 6A). The calibration plots showed good performance between the nomogram prediction and actual observation in terms of the 1-, 3- and 5-year survival rates in TCGA cohort (Figure 6B). The nomogram also showed a favorable predictive ability for 5-year survival rates, with a high AUC value of 0.802 (Figure 6C). These results suggest that the established nomogram could be a reliable and clinically applicable method for predicting the prognosis of melanoma patients.

## Discussion

Malignant melanoma is the most aggressive malignant skin cancer and one of the leading causes of skin cancer-related mortality worldwide. Although bulk RNA transcriptomic data have provided valuable insights into the biological processes of treatment responses, such classic approaches only detect a limited number of analytes in the assay, which reduces the power to characterize the diversity of cellular subtypes and molecular states. In our study, we identified molecular patterns that were co-expressed in melanoma based on NMF and further divided them into six subsets in melanoma, which showed different characteristics concerning the cell cycle, biological functions, and TF network. We also analyzed the relationship between different tumor subtypes and prognosis and found that the cell cycling cluster was associated with poor prognosis. We also identified that the Wnt signaling-enriched cluster may contribute to immunotherapy resistance. In addition, based on the bulk RNA-seq data, we established a prognostic model to classify different risk groups and predict the clinical outcomes of melanoma patients. The results obtained in this study may deepen the understanding of tumor cell subtypes and their relationship with prognosis and drug therapy, thus improving current targeted therapy.

First, we identified tumor cells by inferred large-scale CNVs from single-cell expression profiles and newly identified PMEL, S100B, SERPINE2, TYR, and PRAME as marker genes for melanoma malignant cells. These genes were involved in tumor formation and development in melanoma. For example, PMEL is a specific marker for melanoma with low expression in other tissues. It was crucial for the melanosomal fibril formation through the transition from stage I to stage II melanosomes and overexpressed in more than 75% of human melanomas (Zhang et al., 2021). PMEL has been a target antigen in adoptive T-cell therapy and has been proven to have safety and effectiveness (Johnson et al., 2009). S100B is the marker for melanoma malignant cells and has been shown to interact with p53 in a negative feedback loop (Lin et al., 2010). S100B protein served as a well-analyzed biomarker in melanoma and performed well in detecting early disease progression in high-risk melanoma patients (Ertekin et al., 2020). SERPINE2 was critical for melanoma invasion and correlated with tumor progression (Perego et al., 2018). SERPINE2 could also promote melanoma metastasis through the glycogen synthesis kinase 3β (GSK-3β) signaling pathway (Wu, 2016). TYR encodes tyrosinase, which is responsible for the first step in melanin production. Mutations in TYR can result in the production of abnormal proteins and increase melanoma risk (Gudbjartsson et al., 2008). PRAME is also a tumor-associated antigen and has been a promising immunohistochemical marker in melanoma (Lezcano et al., 2018). Therefore, the malignant marker genes identified in our study may be potential diagnostic markers to distinguish benign from malignant primary cutaneous melanocytic lesions.

Recent single-cell-based studies of tumor cells have discovered new cellular subsets, unique transcriptional programs, and more evidence for “intra-tumoral” and “inter-tumoral” heterogeneity, all of which impact our understanding of therapeutic response and resistance (Patel et al., 2014; Wang et al., 2021). Melanoma patients showed a high degree of ITH in terms of transcriptional programs and CNVs. There is an urgent need for a more precise molecular classification and stratification of melanoma. Tirosh et al. (2016) first uncovered the intra- and interindividual, spatial, functional, and genomic heterogeneity in melanoma cells and identified a highly proliferative cell state linked to resistance to targeted therapies. We also identified the cell cycling subset in melanoma and supplemented more substantial information. Our results showed that cluster 1 had high expression of cell proliferation genes and low expression of checkpoint molecules and antigen-presenting molecules, suggesting poor prognosis. In immunotherapy-resistant patients, the increased proportion of cluster 1 may be an important factor in the relapse of the disease. Cluster 1 also had unique TF and cellular metabolic processes. E2F7, E2F1, MYBL2, and SMC3 were identified as the unique TFs in cluster 1. E2F7 proved to promote cell proliferation, cell cycle progression, cell metastasis, and tumorigenicity abilities (Yang et al., 2020). E2F1 played a major role in the control of the cell cycle under physiological and pathological conditions and was highly expressed in melanoma cells. E2F1 was also associated with the resistance of melanoma cells to BRAF inhibitors, suggesting that targeting the E2F1 signaling pathway may be therapeutically relevant (Rouaud et al., 2018).

In addition to the cell cycling cluster, we also identified five clusters of malignant cells in melanoma. Malignant cell clusters varied significantly in biological characteristics, implying that they may respond differently to therapies. For example, cluster 3 had high expression of immune checkpoint molecules and they were downregulated after ICI treatment. Patients with a high proportion of cluster 3 may be able to respond to immunotherapy effectively. cluster 5 was enriched in Wnt signaling, which help melanocytes bypass senescence and start to proliferate (Gajos-Michniewicz and Czyz, 2020). Cluster 5 signature also indicated a poor prognosis. Wnt signaling was also correlated with immune exclusion in melanoma by reducing the secretion of CCL4, a chemokine that attracts the immune cells (Spranger et al., 2015; Weppler et al., 2020) It was also involved in melanoma progression by regulating cell proliferation and invasion and promoting resistance to targeted therapies (Xue et al., 2016). Thus, we observed a high resistance score in cluster 5 and an increased proportion of cluster 5 after ICI therapy. The combination of Wnt signaling inhibitors and ICI treatment could be a potentially effective treatment for non-responders in melanoma.

The immune microenvironment plays a role in response and resistance to ICI therapy in melanoma. Our work also identified the role of immune and stromal clusters in immune therapy. We found that CD8^+^ T cells played a major role in antitumor immunity and had the lowest resistance score. They have been shown to be predictive biomarkers for response to ICI in melanoma patients (Subrahmanyam et al., 2018). Furthermore, T-cell states, including signatures of IFN-γ responses and those of T-cell activation, exhaustion, and cytotoxicity, have been reported in several studies (Ayers et al., 2017; Prat et al., 2017; Riaz et al., 2017). B cells also have a low resistance score and contribute to antitumor responses. B cells localized in the so-called tertiary lymphoid structures (TLSs) were reported to be associated with improved prognosis and immunotherapy by improving antigen presentation, increasing cytokine-mediated signaling, and releasing tumor-specific antibodies (Cabrita et al., 2020). It was found in our study that fibroblasts had effects on resistance to immune therapy. Cancer-associated fibroblasts (CAFs) can inhibit both the innate and adaptive antitumor immune response by secreting numerous chemokines and cytokines, such as TGF-β, IL-6, IL-8, IL-13, CXCL12, CXCL14, and VEGFA. Furthermore, CAFs synthesize the extracellular matrix (ECM) components such as collagen, fibronectin, and matrix metalloproteinases (MMPs), contributing to increased ECM stiffness, which in turn reduces the infiltration of effector T cells (Liu et al., 2019). Thus, CAFs may serve as an emerging target of anticancer immunotherapy.

It was found that the malignant genes were closely associated with the immune microenvironment of melanoma. We performed consensus clustering based on malignant gene expressions and divided the training cohort into 3 clusters and found patients in different clusters had different clinical outcomes and immune microenvironments. Our survival analysis revealed that patients in cluster 2 had a favorable survival. In addition, the immune and stroma scores and immune cell infiltration were higher in cluster 1. Patients in cluster 3 showed unfavorable survival with less immune cell infiltration. We further constructed a risk model to explore and validate the association between metagenes and the survival state and used PSME2, ARID5A, SERPINE2, CPC3, and S100A1 to construct a risk signature. PSME2 not only balanced proteasome function but also correlated with multiple malignancies and acted as prognostic predictors (Wang et al., 2019). ARID5A was a dynamic molecule that was translocated to the cytoplasm and stabilizes a variety of inflammatory mRNA transcripts, including IL-6, STAT3, OX40, T-bet, and IL-17-induced targets, and contributes to the inflammatory response (Nyati et al., 2019). ARID5A may induce immune cell infiltration and benefit prognosis (Nyati et al., 2019). SERPINE2 promoted melanoma metastasis through the glycogen synthesis kinase 3β (GSK-3β) signaling pathway in a mouse model (Wu, 2016). However, we found that SERPINE2 was associated with favorable prognosis. Therefore, its role in melanoma needs further exploration. S100A11 could promote proliferation, migration, and invasion of tumor cells in multiple cancers (Anania et al., 2013). Based on these significant genes, we constructed a risk model and found it had good performance in predicting survival statistics in TCGA cohort and validation cohort with higher AUC values than the model constructed by Huang et al. (2020) and Ju et al. (2021). In addition, our multivariate Cox analysis showed that the risk score was an independent risk factor for melanoma patients, which was not affected by other factors such as age and gender. In addition, we combined the routine clinical factors associated with OS to construct a nomogram model for clinical application. The calibration curves for OS at 1-, 3-, and 5-year OS demonstrated good agreement between prediction and observation.

In conclusion, based on an integrated analysis of bulk and single-cell expression data, we comprehensively explored the transcriptional characteristics of melanoma subsets and conducted a novel prognostic model to clarify different risk groups of melanoma patients, which may help in planning individualized treatment and improving clinical outcomes.

## Materials and Methods

### Data Sources

The scRNA-seq data were obtained from Jerby-Arnon et al. (2018) containing 31 malignant melanoma patients. Among them, 15 patients were untreated, 15 patients were post-immunotherapy-resistant, and one patient was a post-immunotherapy responder. For the development of the risk score signature, we analyzed the transcriptome-level gene expression from TCGA SKCM dataset (https://portal.gdc.cancer.gov/). For the validation of the identified risk score outcome signature, we further analyzed a large public gene expression dataset from GSE65904 (Cabrita et al., 2020). Immunotherapy data of melanoma were obtained from GSE91061 (Riaz et al., 2017), containing 23 partial response/complete response (PR/CR) patients and 48 progressive disease (PD) patients. Clinical data for included patients could be obtained in Supplementary Table S1.

### Single-Cell RNA Data Analysis

Single-cell gene expression counts were analyzed by the “Seurat” package (version 3.99) (Stuart et al., 2019). Single cells with less than 200 unique molecular identifiers (UMIs) or with more than 20% mitochondrion-derived UMI counts were considered low-quality cells and removed. For the remaining high-quality cells, gene expression matrices were normalized using the “NormalizeData” function and scaled with the “ScaleData” function to gain linear conversion. Top 2000 variable genes were extracted to perform the principal component analysis (PCA), and the 30 top significant principal components were used for cluster analysis. Then single-cell data from different samples were then integrated, and the batch effects were removed by using the “Runharmony” function in the Harmony package (Korsunsky et al., 2019). Uniform Manifold Approximation and Projection (UMAP) was used for the visualization of the clusters. The tumor/normal prediction was performed by CopyKAT (Gao et al., 2021) by the default parameters. The predicted aneuploid cells were inferred as tumor cells, and diploid cells as normal cells. Other cell types were annotated based on the expression of known markers such as T cells (CD3D, CD4, and CD8), NK cells (NKG7 and FGFBP2), myeloid cells (CD14, CD68, and CD1A), B/plasma cells (CD79A, MS4A1, and MZB1), fibroblasts (DCN and ISLR), endothelial cells (VWF and PECAM1), and pDCs (CLEC4C and LILRA4). The “FindAllMarkers” function was used to calculate the markers of each cell cluster, with a threshold of log_2_FC > 0.25 and min.pct>0.25.

### Correlation Analysis Between Clusters

To explore the correlation of clusters, we calculated the Spearman correlation coefficient between two clusters by using the “corr. Test” function in the “Psych” package according to the expression levels of top 30 marker genes. The correlation coefficients between cell subsets are shown in the heatmap.

### Cell Cycle Analysis

Knowing that the cell cycle plays an important role in tumor progression, we used the “CellCycleScoring” function in Seurat to calculate the cell cycle score of each cell. The cell cycle phase marker genes for humans to perform phase scoring were based on Tirosh et al. (2016). Then we categorized them into different stages according to the phase scoring. The results are expressed in the bar plot to show the proportion of the cell cycle in different clusters or subgroups.

### Copy Number Variation Analysis

We used “inferCNV” (https://github.com/broadinstitute/inferCNV/wiki) to estimate copy number variations by the expression levels of genes within each chromosome region. The UMI count matrix was extracted from Seurat to prepare the input file. High-quality cells with at least 5 genes in each chromosome were calculated for DNA copy numbers. Endothelial cells, fibroblasts, and myeloid cells were also calculated as a CNV control. To normalize the CNV profiles, we then subtracted the average expression profiles of the normal sample from the entire CNV dataset. Cells with deletions and amplification of entire chromosomes were visualized in a heatmap.

### NMF Identification of Intra-Tumoral Transcriptional Heterogeneity

NMF was used to identify variably expressed metagenes across melanoma patients using the “NMF” R package (version 0.20.6) (Gaujoux and Seoighe, 2010). They were then compared by hierarchical clustering, using one minus the Pearson correlation coefficient over all gene scores as a distance metric. Six clusters of signatures were identified manually. For each signature, we further performed Gene Ontology (GO) and KEGG analysis using the “clusterprofiler” package and calculated the mean loadings for each of the top 30 genes. Genes with the highest loading were defined as the marker genes for the signature.

### Signature Score Calculation

The expression of a gene signature in each patient was evaluated by using a single-sample gene set enrichment analysis (GSVA) (Hänzelmann et al., 2013). To assess the prognostic values of the gene signatures, patients in TCGA SKCM cohorts were allocated into high- and low-expression groups according to the median value of the GSVA score for each signature gene set. Kaplan–Meier survival curves were plotted to show differences in survival time with the R “Survival” package.

### TF Analysis

In order to further study the interaction mechanism between cell subpopulations, we used the SCENIC (Aibar et al., 2017) to calculate the TF regulatory network between related cell subpopulations. Briefly, SCENIC included three steps: First, we conducted GRNboost to identify co-expression modules between TFs and the potential target genes; second, cisTarget was used for each co-expression module to infer direct target genes based on the significantly enriched motif of the corresponding TF is; and third, we conducted AUCell to calculate cellular regulon enrichment scores through the AUC. The specific TF was calculated in accordance with Suo et al. (2018). Regulon specificity score (RSS) was calculated by the “calcRSS” function in the SCENIC. The RSS is calculated for each cell cluster separately, and the top five regulons are shown.

### DEGs and GO Analysis

DEGs between non-responders and responders in pre-therapy and on-therapy groups were identified using the “Limma” package. The significantly upregulated and downregulated genes (*p* < 0.05, |logFC|>1) were subjected to GO analysis using the clusterProfiler 4.0. (Wu et al., 2021). The GO network plots were built using the “cnetplot” function in the “enrichplot” R package.

### Resistance Score Calculation

DEGs between non-responders and responders were included as the input gene set. Then we utilized the “AddModuleScore” function in Seurat to calculate the scores of the upregulated genes and the downregulated genes. The resistance score was calculated by scores of upregulated genes minus the downregulated genes.

### Consensus Clustering

We applied the “ConsensusClusterPlus” R package to categorize melanoma patients in TCGA dataset into different groups (Wilkerson and Hayes, 2010). We selected 90% item resampling (pItem), 100% gene resampling (pFeature), and a maximum evaluated k of 9 and 100 re-samplings (reps) with agglomerative hierarchical clustering upon Euclidean correlation distances to partition patients.

### Function Analysis of Subgroups and Immune Infiltration Analysis Based on ssGSEA

GSVA was performed to evaluate pathway enrichment for different clusters with the R package “GSVA.” To investigate the immune infiltration signature of melanoma, ssGSEA was performed to assess the level of immune infiltration based on the expression levels of immune cell-specific marker genes. We also performed a functional analysis of the clusters based on the hallmark gene set from the MsigDB dataset (https://www.gsea-msigdb.org).

### Construction and Evaluation of the Prognostic Risk Score Model for Melanoma

We first performed univariate Cox regression analysis on TCGA SKCM training cohort and identified the association between the expression levels of the genes and survival time with the survival package. Significant genes (*p* < 0.05) identified by univariate Cox regression were further selected by lasso Cox regression and multivariate Cox regression. The 5 optimal genes were used to construct a prognostic risk score model by using the following formula: risk score = ∑Coefi·Expi. According to the median value of their prognostic risk scores, patients were subgrouped into a high-risk group and a low-risk group. The Kaplan–Meier survival curve was used to assess the differences in OS between the two groups. Melanoma patients with the R package “survival” and time-dependent ROC curve data were used to evaluate the prognostic performance of the constructed risk model.

### Statistical Analysis

Statistical analyses were mainly performed using R (version 4.0.4) and GraphPad Prism (version 8.0.1). Kaplan–Meier and log-rank analyses were used to evaluate the survival differences between different groups of patients. Student’s t-test and one-way ANOVA were used to estimate the differences between two groups and more than two groups. The correlation analysis was calculated using the “Spearman” method. Two-sided *p* < 0.05 was regarded as statistically significant.

## Data Availability

Publicly available datasets could be found in GEO dataset (https://www.ncbi.nlm.nih.gov/geo/, accession code GSE119578, GSE69054 and GSE91061) and TCGA-SKCM dataset (https://portal.gdc.cancer.gov/projects/TCGA-SKCM.)

## Supplementary Material

Supplementary Figure S1 Feature plots of the expression levels of representative marker genes of the melanoma clusters. The color represents the gene expression levels.

Supplementary Figure S2 Heatmap of large-scale CNVs of tumor subsets from melanoma patients. The heatmap shows the normalized CNV levels; the red color represents high CNV levels; and blue represents low CNV level. CNV: copy number variation.

Supplementary Figure S3 GO and KEGG analysis of malignant signatures. **(A,B)** Bar plot shows the enriched GO term and KEGG pathways in six malignant signatures according to the gene counts (x axis) and p values (color). **(C)** UMAP plots of tumor subsets split by treatment. **(D)** Dot plot displaying the fractions of expressing cells (dot size) and mean expression level in expressing cells (dot color) of marker genes (rows) across clusters.

Supplementary Figure S4 Boxplots showing the expression levels of cell cycle genes, immune checkpoint genes and antigen presentation genes between no-treatment and immunotherapy groups.

Supplementary Figure S5 Subgroups of melanoma patients in TCGA SKCM. **(A)** Consensus clustering matrix showing the melanoma patients in TCGA SKCM were clustered into 3 subgroups by ConsensusClusterPlus based on metagenes. **(B)** Kaplan–Meier curves for progression-free survival according to patient clusters evaluated by ConsensusClusterPlus. **(C)** Heatmap of immune infiltration scores of TCGA-SKCM among subgroups. **(D)** Heatmap of the biological function scored by ssGSEA among subgroups.
