# Supplementary material for: Identification of Transcriptional Heterogeneity and Construction of a Prognostic Model for Melanoma Based on Single-Cell and Bulk Transcriptome Analysis
Source: Front Cell Dev Biol. 2022 May 13;10:874429. doi: 10.3389/fcell.2022.874429 (PMC9136400; doi:10.3389/fcell.2022.874429)
Supplement: Supplementary file 5 [file DataSheet1.docx]

Supplementary Material

## Supplementary Figures


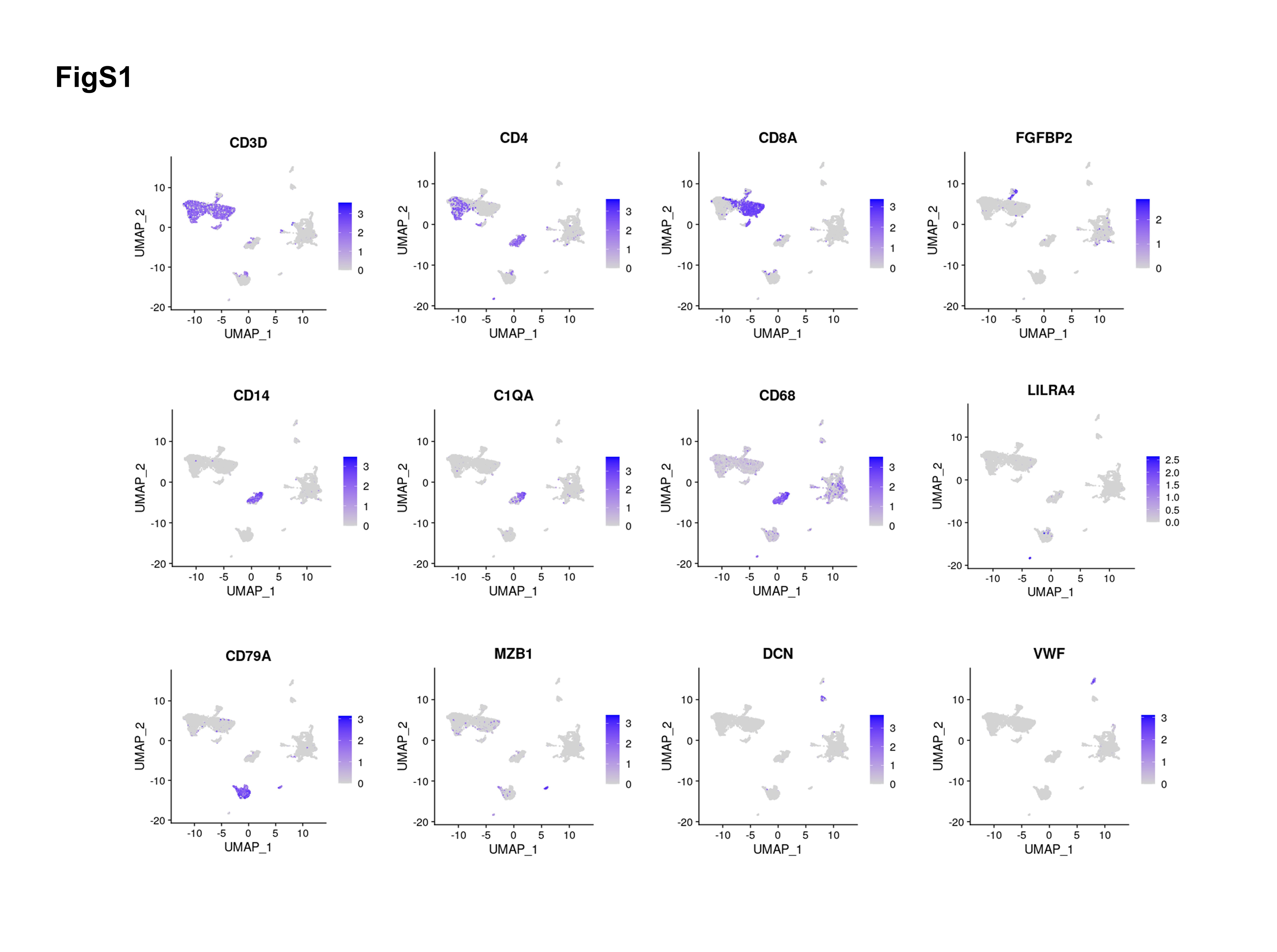


**Supplementary Figure 1.** Feature plots of the expression levels of representative marker genes of the melanoma clusters. The color represents the gene expression levels.


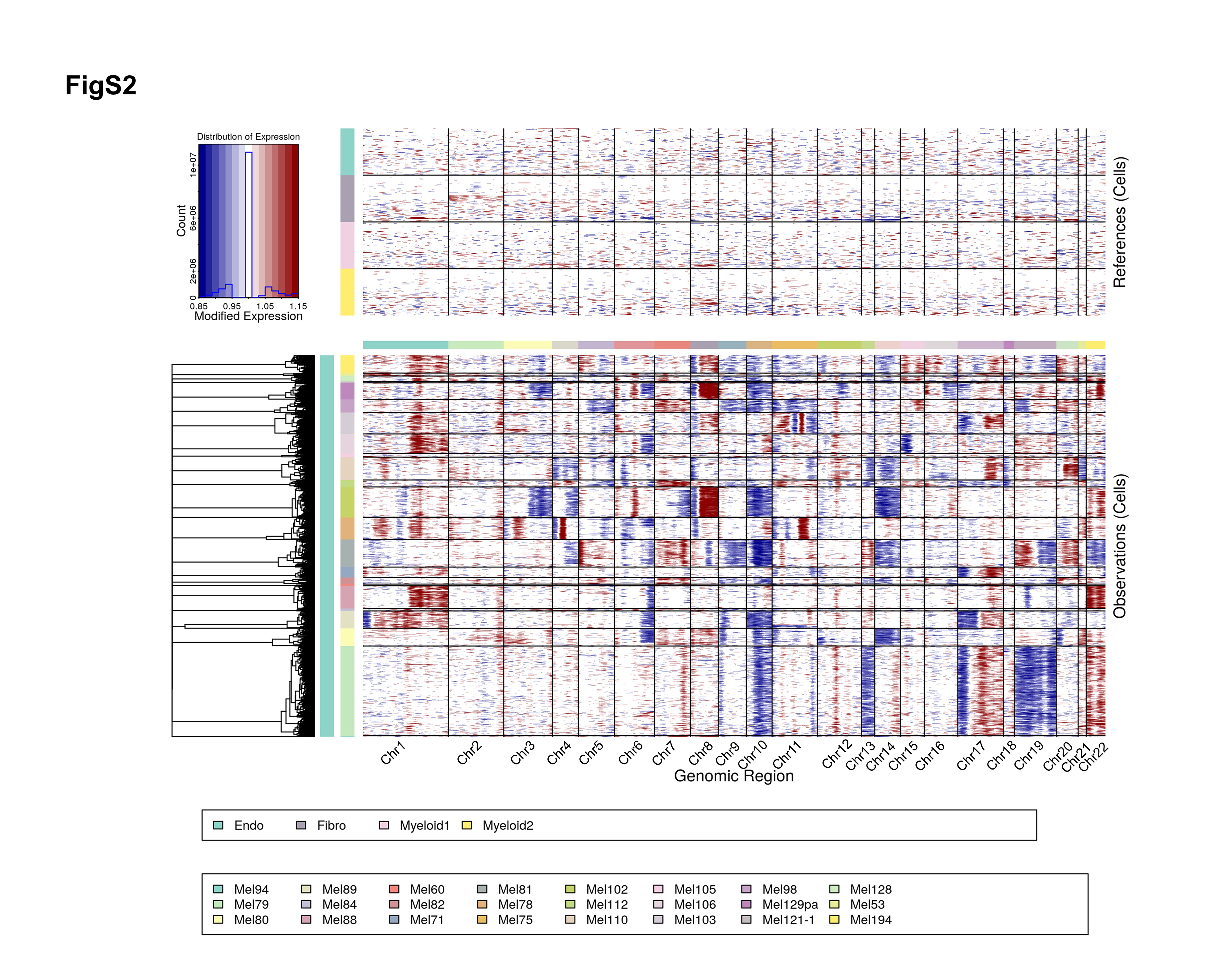


**Supplementary Figure 2.** Heatmap of large-scale CNVs of tumor subsets from melanoma patients. The heatmap shows the normalized CNV levels; the red color represents high CNV levels; and blue represents low CNV level. CNV: copy number variation.


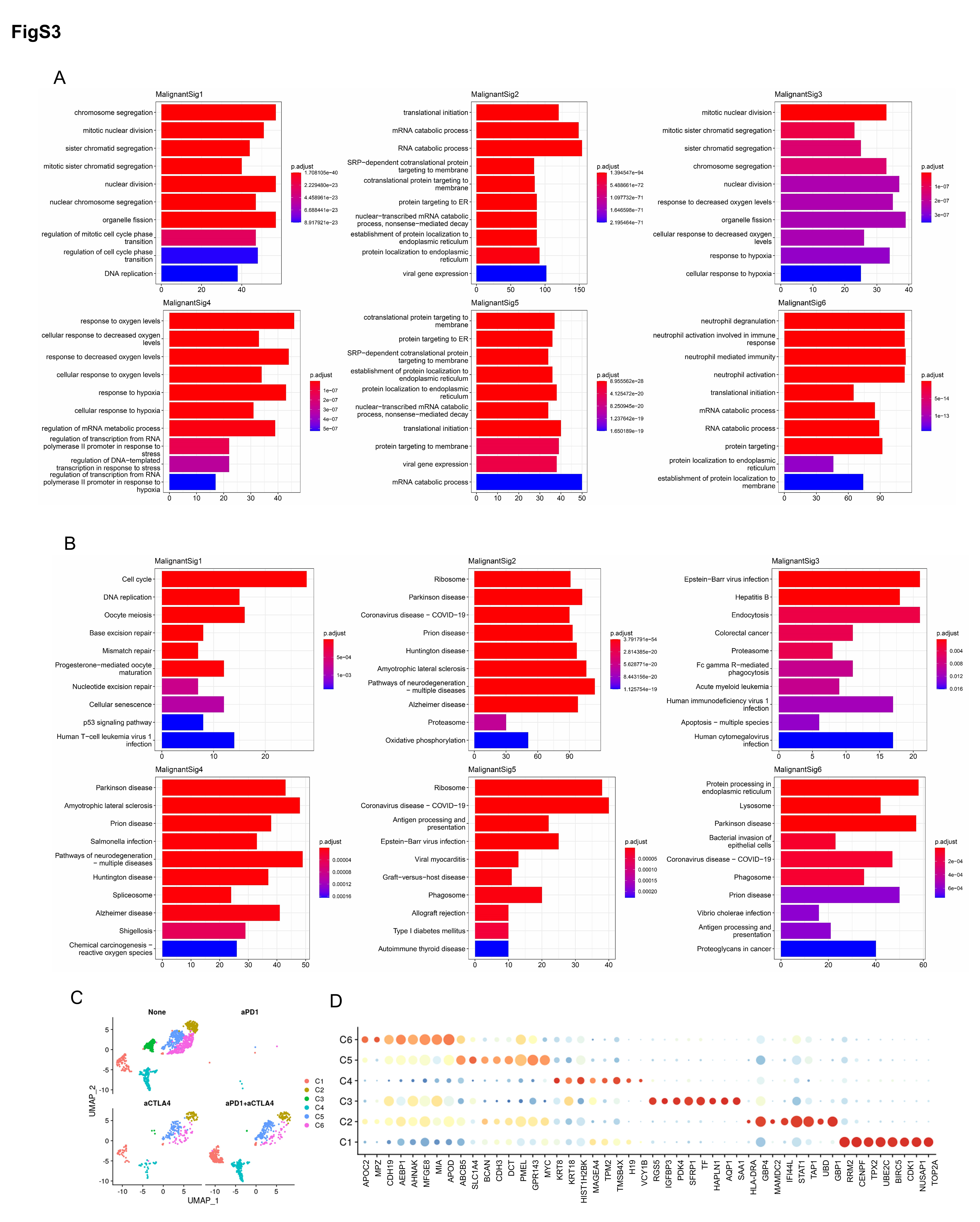


**Supplementary Figure 3.** GO and KEGG analysis of malignant signatures. (A-B) Bar plot shows the enriched GO term and KEGG pathways in six malignant signatures according to the gene counts (x axis) and p values(color). (C) UMAP plots of tumor subsets split by treatment. (D) Dot plot displaying the fractions of expressing cells (dot size) and mean expression level in expressing cells (dot color) of marker genes (rows) across clusters.


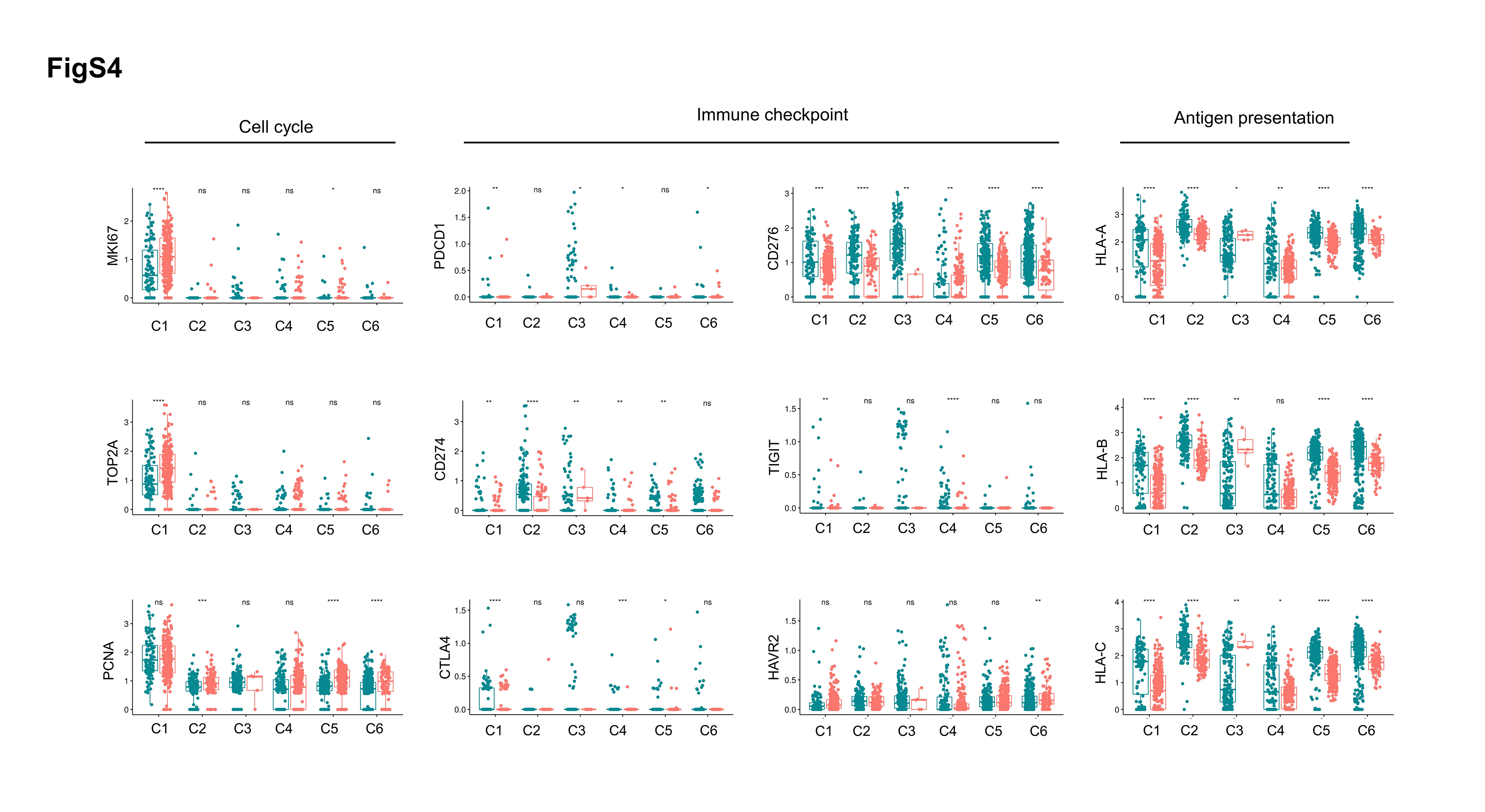


**Supplementary Figure 4.** Boxplots showing the expression levels of cell cycle genes, immune checkpoint genes and antigen presentation genes between no-treatment and immunotherapy groups.


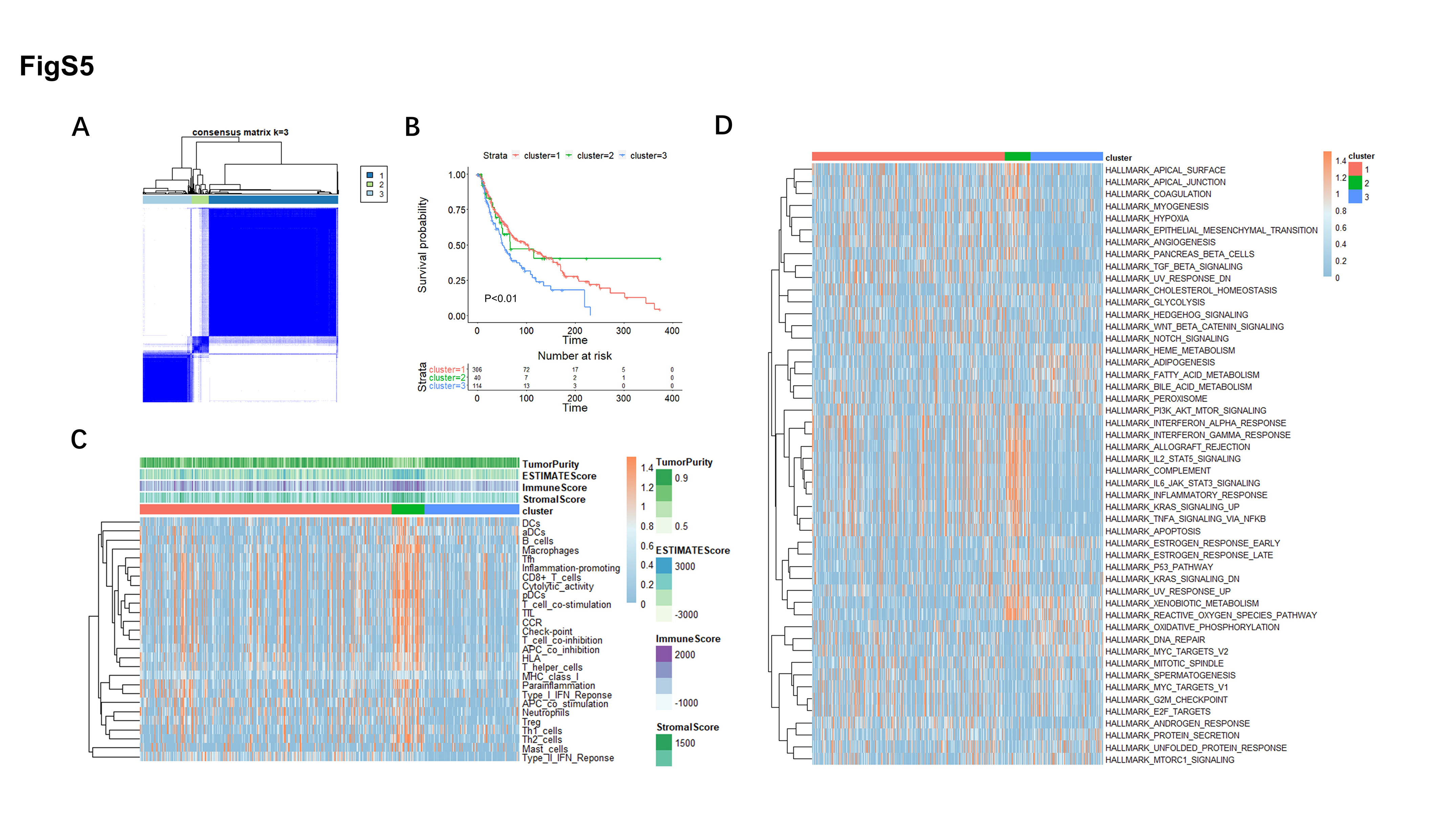


**Supplementary Figure 5. Subgroups of melanoma patients in TCGA SKCM.** (A) Consensus clustering matrix showing the melanoma patients in TCGA SKCM were clustered into 3 subgroups by ConsensusClusterPlus based on metagenes. (B) Kaplan–Meier curves for progression-free survival according to patient clusters evaluated by ConsensusClusterPlus. (C) Heatmap of immune infiltration scores of TCGA-SKCM among subgroups. (D) Heatmap of the biological function scored by ssGSEA among subgroups.
